# Equity in out-of-pocket payment in Chile

**DOI:** 10.1590/S1518-8787.2017051006666

**Published:** 2017-04-24

**Authors:** Alicia Lorena Núñez Mondaca, Chunhuei Chi

**Affiliations:** I Department of Management Control and Information Systems. School of Economics and Business. University of Chile. Santiago, Chile; II Center for Global Health. International Health Program. College of Public Health and Human Sciences. Oregon State University. Corvallis, Oregon, United States

**Keywords:** Health Care Costs, Health Equity, Equity in Access, Health Services Accessibility, Health Systems, economics

## Abstract

**OBJECTIVE:**

To assess the distribution of financial burden in Chile, with a focus on the burden and progressivity of out-of-pocket payment.

**METHODS:**

Based on the principle of ability to pay, we explore factors that contribute to inequities in the health system finance and issues about the burden of out-of-pocket payment, as well as the progressivity and redistributive effect of out-of-pocket payment in Chile. Our analysis is based on data from the 2006 National Survey on Satisfaction and Out-of-Pocket Payments.

**RESULTS:**

Results from this study indicate evidence of inequity, in spite of the progressivity of the healthcare system. Our analysis also identifies relevant policy variables such as education, insurance system, and method of payment that should be taken into consideration in the ongoing debates and research in improving the Chilean system.

**CONCLUSIONS:**

In order to reduce the detected disparities among income groups, healthcare priorities should target low-income groups. Furthermore, policies should explore changes in the access to education and its impact on equity.

## INTRODUCTION

Inequity in the financial burden of the healthcare is a major concern worldwide, either in high income or low income countries. To better understand this idea, according to Starfield^14^, equity in health services “implies that there are no differences in health services where health needs are equal (horizontal equity) or that enhanced health services are provided where greater health needs are present (vertical equity)” (p.546)^14^. The World Health Organization^a^ considers as inequity the differences in health status that are unnecessary, avoidable, unfair, and unjust. Therefore, equity can be referred as social justice about the things that a person has as a right^21^. The main equity objective is to create opportunities to provide equal health for all, affecting, among other things, health outcomes, and utilization of and access to healthcare services^23^. While issues of equity in health system finance have been well studied in member countries of the Organization for Economic Cooperation and Development (OECD), there are still few published empirical studies on Latin-American healthcare systems, where a wider gap in income and wealth distribution exists within Governments. Even though Chile is now part of the OECD, it has the widest gap between rich and poor among the 34 OECD countries, indicating the higher financial burden of out-of-pocket (OOP) in healthcare for persons in lower income groups.

The healthcare system in Chile is mixed, both in the financing and delivery of services. Citizens can choose between public or private health insurance. The National Health Fund (FONASA) is a public organization, which is divided into four levels of income groups (A, B, C, and D) and provides coverage to 72% of the population, without discrimination of age, gender, income level, number of dependents, or pre-existing legal conditions^b^. Private health insurances, ISAPRE hereafter, are private institutions that provide services to approximately 16% of the Chilean population^c^. These two systems share a common financing source: the obligatory contribution of employees, which is a 7% monthly income tax with a limit of 74,3UF (unit of account used in Chile), equivalent to US$2,703.55^d^ ^,^ ^e^. Even though most of the Chilean population is covered by a health insurance, reaching effective universal coverage^13^, there are differences within and between each type of insurance and the health plans provided depending on income level, i.e., more money means better health care plans. To solve this issue, previous governmental initiatives have attempted to improve equity in the Chilean healthcare system. From 1990 to 2000, two social policies were developed, the first guaranteeing an adequate level of healthcare services based on an equal opportunity perspective, and the second focused on specific programs directed to disadvantaged groups to promote access to the opportunities available at that time^18^. These social policies, which were aimed at improving equity in healthcare in terms of access and lower financial burden for the low income family, were successfully implemented achieving the short-term objectives with the proliferation of new programs. However, these policies were unable to address major social issues, such as poverty, which is accompanied by drug consumption, violence, insecurity, and others^12^. Between 2000 and 2006, the proposed programs were focused on the improvement of social determinants of health. Between 2003 and 2004, a new legal framework of health was approved, known as the Regime of Explicit Health Guarantees, which is a program that brings a legal provision of social guarantees that defines the principles of access, quality, opportunity, and financial protection.

Based on the healthcare reforms in Chile, previous studies have attempted to better understand the state of health equity in Chile. A study promoted by a governmental initiative and carried out by Jadue and Marín^5^ has focused on health equity issues with the objective of identifying social determinants of health in Chile. Also, the EQUILAC project, from the Pan American Health Organization, is looking for a systematic assessment of equity in health systems in the Latin American and the Caribbean region, in which we can find some recent studies about equity in health and healthcare utilization in Chile^17^. Specifically in health system finance, Cid and Prieto^3^ have examined the evolution of OOP payment in Chile using the Survey of Family Budgets based on the years 1997 and 2007 for the capital of Chile, Santiago. The main findings are the growth of household spending – more than 39.5% *per capita* – and the gap that persists between the proportion of OOP expenditures and the total expenditures between the richest and the poorest households. One interesting fact about their study is that it compares the two years before and after the implementation of the Explicit Health Guarantees (former AUGE [Universal Access to Explicit Health Guarantees]) program; however, they show that even though the program has had an effect in the containment of OOP spending, new financial protection policies are required. Following these lines, another study conducted by Bitrán and Muñoz^2^ uses the 2006 Satisfaction and Out-of-Pocket Payment Survey to identify health expenses inadequately covered by current insurance plans. The authors have found that almost half of the healthcare expenditures are paid directly by household OOP, being supplies and medications the most important sources of OOP spending, given the existing insurance coverage. This information is supported in the study conducted by Urriola^15^ about the way the healthcare system is financed in Chile. The study has shown that the first income quintile pays 68% of its OOP spending in medications, while the fifth quintile pays 43% of its OOP spending in medications. Bitrán and Muñoz^2^ have also found that OOP expenditures increase with income.

As shown above, there are very little empirical studies on equity in health finance in Chile. Therefore, the purpose of this study is to expand this area of research and assess the distribution of financial burden in Chile, with a focus on the burden and progressivity of OOP payment, based on the principle of ability-to-pay (ATP). Factors that contribute to inequities in health system finance were explored, along with possible policy variables that may contribute to the more equitable distribution of financial burden in healthcare.

## METHODS

The data for this study were drawn from the 2006 Satisfaction and Out-of-Pocket Payment Survey. This survey uses representative random sample from the national population. There are no new versions of this survey. The sample design is strictly probabilistic, multistage, including geographical stratification and conglomerates. The sample of the survey involves 4,558 households or 16,519 people.

Using this data, we considered two different alternatives to examine financial equity issues. The starting point involves the use of a two-part model (2PM) to estimate factors associated with financial burden of healthcare in Chile, allowing us to detect the presence of inequity. This model was selected because of the large number of non-users of health care services, and to allow a better performance of the model avoiding the excess of zeros in the analysis of OOP payment. Moreover, to have a comprehensive understanding of the distribution of financial burden, we used an additional method of measurement. The second method includes the computation of progressivity indexes, which allowed us to estimate how much higher OOP payments are as a share of the ATP for the poor than for the rich. Both methods complement each other in providing evidence of inequity.

The final objective is twofold: to estimate the amount of OOP payment for healthcare and to assess the factors associated with it under the Chilean healthcare system. This was motivated by the concern of financial burden in Chile, where new public health programs have been recently introduced (such as Explicit Health Guarantees), and the role of the country as one of the 12 members of the Global Alliance of Health Equity. The significance of this study is that, in system undergoing changes and becoming more publicly financed, persons may increasingly value the legitimacy of the public system. Furthermore, high-income households may see public finance as a financially attractive alternative and either oppose any increase, or even support the reduction, in privately financed healthcare. This concern is based on a normative assumption: a progressive publicly financed healthcare system is a preferred system for its universality and in line with the ATP principle^19^. Moreover, as OOP payments for healthcare services increase, the financial burden of healthcare tends to become more regressive, thus placing a heavier burden on low-income families^20^.

Thus, to carry out this study we selected a group of variables from the 2006 Satisfaction and Out-of-Pocket Payment Survey including a dichotomous indicator of whether a person has health care visits during the year of analysis, as well as a set of sociodemographic variables of the participants such as age in years, gender , marital status, education level (ranging from no education, be studying or having a degree offered by a technical school, up to be studying or having a college degree), the region where the participants live, work status, income, and also household expenditures on food, transportation, education and recreation, housing, clothing, and healthcare. In addition, we have included variables to assess whether the individual has a healthcare insurance or not, and the type of insurance: Indigent card, FONASA (public insurance), ISAPRE (private insurance), CAPREDENA (National Defense Pension Fund), DIPRECA (National Insurance for the Police), other system, or no insurance at all, and finally we also have a variable to assess if the individuals had an additional health insurance. A summary of key variables and exploratory data analysis from the 2006 Survey are presented in Table 1, which describes the measurement scales and descriptive statistics.

**Table 1:** Variables selected from the 2006 Satisfaction and Out-of-Pocket Payment Survey. Chile, 2006.

Variable	Measurement scale	Definition	Mean	SD	Min	Max
Health care	Nominal/Discrete	Health care visits: 0 = no, 1 = yes	0.410	0.491	0	1
Age	Interval/Discrete	Age in years at the time of the survey (18 to 98)	52.380	15.314	18	98
Gender	Nominal/Discrete	Gender: 0 = male and 1 = female	0.317	0.465	0	1
Marital status	Nominal/Discrete	Marital status: married, living with partner, annulment /separation/divorce, widowed, and single	2.148	1.462	1	5
School	Nominal/Discrete	Last level of study approved: no education, elementary school, high school, technical-professional school, technical training center, professional institute, university	4.483	1.767	1	7
Region	Nominal/Discrete	II region, V region, VIII region, or XIII region (Metropolitan region)	9.091	3.819	2	13
Work status	Nominal/Discrete	Did you work last week? 0 = no, 1 = yes	0.633	0.481	0	1
Ln(income)	Interval/Continuous	Individual’s level of income (0 to 15.90 Chilean pesos)	11.703	2.470	0	15.899
Ln(food)	Interval/Continuous	Household food expenditures (0 to 13.49 Chilean pesos)	11.367	0.845	0	13.487
Ln(transportation)	Interval/Continuous	Household transportation expenditures (0 to 14.26)	9.267	3.017	0	14.262
Ln(education)	Interval/Continuous	Household education and recreation expenditures (0 to 15.27)	5.654	5.147	0	15.267
Ln(living)	Interval/Continuous	Household living expenditures (0 to 15.65)	11.024	1.237	0	15.652
Ln(clothing)	Interval/Continuous	Household clothing expenditures (0 to 13.63)	7.967	3.486	0	13.629
Ln(other)	Interval/Continuous	Other household expenditures (0 to 15.04)	5.925	4.746	0	15.039
Ln(OOP)	Interval/Continuous	Out-of-pocket expenditures (0 to 13.31)	4.645	4.732	0	13.306
Ins_system	Nominal/Discrete	Do you belong to a health insurance system? 0 = no, 1 = yes	0.723	0.447	0	1
Insurance	Nominal/Discrete	Type of insurance system: Indigent card, FONASA, ISAPRES, CAPREDENA, DIPRECA, other system, no insurance	2.550	1.589	1	7
Dependent	Nominal/Discrete	Are you a dependent worker with compulsory deductions for the payment of health insurance? 0 = no, 1 = yes	0.393	0.488	0	1
Additional benefits	Nominal/Discrete	Do you pay in addition to the 7% of your taxable salary for health insurance? 1 = yes, 2 = no, 3 = do not apply	2.300	0.623	1	3
Emergency insurance	Nominal/Discrete	Do you have an emergency insurance? 0 = no, 1 = yes	0.024	0.154	0	1
Additional insurance	Nominal/Discrete	Do you have an additional insurance? 0 = no, 1 = yes	0.063	0.244	0	1

Ln: Natural Logarithm; Ins: Insurance

As we mentioned before, we have used two different methods for the data analysis. The first is the 2PM, where an individual’s OOP payment for 2006 was the unit of analysis for our estimation. The OOP payment for healthcare services was expected to suffer a skewed distribution around zero, because of differences between persons not using medical services (0 payment) and those with expensive medical care. To address this issue, we use a 2PM developed by Manning et al.^7^ ^,^ ^9^

The first part of the model involves the estimation of a logit model that describes the distinction between non-users and users of healthcare services. The dependent variable for the logit model is a binary variable (0 = no use of healthcare, and 1 = use of healthcare [having zero or positive expenses]). The second part consists in estimating an ordinary least-square regression for OOP payment for those with non-zero expenses. For the ordinary least-square regression model, the dependent variable is the natural logarithm of OOP payment and the explanatory variables were age, gender, marital status, education, region, work status, insurance system, additional insurance, and the following expenditures (as a proxy measure for wealth or income): food, transportation, education and recreation, and housing and clothing.

Additionally, a progressivity analysis was carried out to quantify inequalities in healthcare expenditure. It measures the deviation between OOP payment and ATP. As ATP increases, we can state that a system is progressive or regressive if healthcare payments account for, respectively, an increasing or decreasing proportion of ATP^24^.

The ATP is measured by an approximation of the household consumption net of non-discretionary expenditures. We applied two methods to assess progressivity of OOP payment: (1) the percentage that OOP payment represents for household expenditures organized by quintile, and (2) the use of concentration curves, comparing shares of health payments contributed by proportions of the population ranked by ATP with their share of ATP.

Therefore, we compared the concentration curve of healthcare payments with the Lorenz curve for ATP^11^. Under a progressive system, the share of health payments for the poor is lower than their share for ATP, and the concentration curve would lie above the Lorenz curve. Under a regressive system, the share of health payments for the poor is greater than their share for ATP, and the concentration curve would lie below the Lorenz curve^11^. The further the concentration curve is from the Lorenz curve, the greater the level of inequity in healthcare payment.

Based on the concentration curve calculation, a concentration index can be estimated, which corresponds to twice the area between the concentration curve and the diagonal. The concentration index can take values between -1 (disproportionate concentration of payment for the poor) and +1 (disproportionate concentration of payment for the rich). A value of 0 means there is no correlation between the share of healthcare payment and ATP^22^.

Kakwani index was also estimated. The index measures twice the area between the Lorenz curve and the concentration curve for health payments. Kakwani progressivity index takes values between -2 to 1. A negative value means that the financial burden is concentrated in the poorest persons; a positive value indicates the opposite; while perfect equality is found with a value of zero.

Finally, to decompose the redistributive effect of healthcare payments we used the formula proposed by Aronson et al.^1^ Healthcare payments in a collective financing system, besides promoting access to healthcare services, can also generate some redistribution of income. The main concern for this redistribution effect comes from the fact that most OOP payments under a collective financing system are unintended or of involuntary nature^22^ ^,^ ^23^. In this sense, the redistribution can be vertical and horizontal. The level of progressivity of the system can help to assess vertical redistribution. The horizontal redistribution occurs when persons with equal ATP make different payments for healthcare^11^. This redistribution effect depends on four factors: the progressivity of the healthcare financing system, the proportion of income allocated to finance healthcare, the degree of horizontal inequity, and the re-ranking among households between the pre- and post-payment income distribution^16^.

We used Stata 11.1 to analyze the data sets for this study. We also used ADePT, a free software developed by the World Bank, to automate and standardize the economic analysis on the equity of financial burden for this research, which includes progressivity and redistribution of the health system finance. The main purpose of using this software was to allow comparability with future studies.

## RESULTS

### Two-part Model

To assess the factors that could explain inequity in health system finance, a 2PM using the 2006 Satisfaction and Out-of-Pocket Payment Survey was estimated (Table 2).

**Table 2:** Two-part model estimation for health care services (selected results).

Variable	Estimation results						

	First part: Logit				Second part: Ordinary least-square		
	
	Equation of probability of use				Equation of expenditures		
	Dependent variable: usage (yes/no)				Dependent variable: Ln(OOP payment)		
	
	Coeff.	SE	z	ME	Coeff.	SE	z
Constant	-4.437	0.673	-6.60^a^		-4.743	1.655	-2.87^a^
Female	0.622	0.099	6.28^a^	0.134	0.381	0.249	1.53
Age	0.023	0.003	7.47^a^	0.050	0.016	0.008	2.10^b^
School							
No education	-				-		
Elementary school	0.206	0.248	0.83	0.042	0.283	0.643	0.44
High school	0.401	0.251	1.59	0.084	1.750	0.653	2.68^a^
Technical-professional	0.290	0.271	1.07	0.060	1.967	0.710	2.77^a^
Technical training	0.242	0.325	0.74	0.050	1.558	0.861	1.81^c^
Professional institute	0.729	0.299	2.44^b^	0.157	2.007	0.775	2.59^a^
University	0.880	0.270	0.26^a^	0.191	2.643	0.692	3.82^a^
Work	-0.471	0.097	-4.87^a^	-0.102	-0.552	0.241	-2.29^b^
Ln(Income)	0.008	0.016	0.51	0.002	0.089	0.041	2.16^b^
Insurance system	0.300	0.109	2.76^a^	0.065	0.646	0.280	2.31^b^
Insurance							
Indigent/FONASA (A, B)	-				-		
FONASA (C, D)	-0.014	0.161	-0.09	-0.003	2.394	0.429	5.58^a^
ISAPRES	0.020	0.204	0.10	0.004	2.329	0.517	4.50^a^
CAPREDENA	0.182	0.377	0.48	-0.071	2.585	0.877	2.95^a^
DIPRECA	-0.327	0.452	-0.72	-0.090	4.609	1.025	4.50^a^
Other system	-0.419	0.680	-0.62	-0.083	-0.311	1.617	-0.19
None	-0.384	0.222	-1.73	-0.042	2.646	0.636	4.16^a^
Emergency insurance	0.676	0.231	2.92^a^	0.146	0.224	0.446	0.50
Additional insurance	0.367	0.143	2.56^b^	0.081	0.466	0.328	1.42
Pseudo-R2/R2	8.24%				21.81%		
Sample	4,269				1,758		

Ln: Natural Logarithm

Note: Z-score is in parentheses and indicates significance level as follows: (^a^) p ≤ 0.01; (^b^) 0.01 < p ≤ 0.05; (^c^) 0.05 < p ≤ 0.10.

The variables statistically significant in the logic model were: gender, age, individuals with education from a professional institute or university, employment status, insurance system, emergency insurance, and additional insurance. The results from the logit model estimation suggested that females were positively associated with the probability of using health services. According to the marginal effect, the probability of using healthcare services for females was 0.134 times higher than for men. Age increased the probability that a person would use healthcare services. For the average person in the sample, an extra year of age increases the probability of usage by 5%. In addition, persons with technical training, or having attended a professional institute, or university showed higher probability of using healthcare services than those with no education.

The variables statistically significant in the ordinary least square model for OOP payment included age, all the categories of the variable school except for no education, employment, natural logarithm of *per capita* income, insurance system, and the following types of insurance: FONASA, ISAPRES, CAPREDENA, DIPRECA, and no insurance. Of those statistically significant variables, the marginal effect of insurance system, school, and income were the strongest. In order to interpret results, the estimated coefficients were exponentiated, and we found that a 1% increase in *per capita* income resulted in increasing OOP payment by 0.09%. Moreover, our estimation also suggests that respondents with high school education had a 475% higher OOP payment than those with no education. This percentage increases as the level of education increases. In addition, there is evidence of inequity in the system. The FONASA users from groups C and D have an increase of OOP payment of 996% than those in groups A and B. Individuals with private insurance have an increase of OOP payment of 927% compared to FONASA groups A and B. Therefore, those with private system are paying a similar or lower OOP payment than those in FONASA groups C and D.

### Progressivity

Table 3 presents the sources of finance by household characteristics. The OOP payments for healthcare services were similar for the II, V, and XIII regions, while it was substantially lower for the VIII region. According to these results, ISAPRE, DIPRECA, and other systems are the insurance systems with the higher household OOP payment.

**Table 3:** Sources of finance by household characteristics (all units are in Chilean Peso).

Variable	*Per capita* consumption, gross	OOP payment for healthcare	*Per capita* consumption, net of payments
Region			
II region	115,671.9	11,951.1	103,720.8
V region	106,632.2	10,647.5	95,984.6
VIII region	81,283.5	8,345.8	72,937.7
XIII region	105,101.1	11,181.7	93,919.4
Insurance			
Indigent card	50,669.1	3,348.6	47,320.5
FONASA	92,565.8	10,416.2	82,149.6
ISAPRE	216,349.9	19,742.8	196,607.1
CAPREDENA	130,159.8	18,958.4	111,201.5
DIPRECA	141,113.7	26,033.7	115,080.1
Other system	278,000.0	26,998.6	251,001.4
None	116,345.4	6,437.6	109,907.8
Missing	95,405.2	10,185.9	85,219.4
Total	100,410.6	10,421.6	89,989.0

Note: 1 US Dollar is equivalent to $463.19 Chilean pesos (Banco central de Chile, September 1, 2011).

To assess progressivity of OOP payment for healthcare, we made a graph on the percentage of total household expenditure by quintile groups (Figure). There is a tendency to increase health payments per household expenditure by quintile groups, indicating some progressivity of the finance system, while this effect is not visible after the third quintile.

**Figure:**
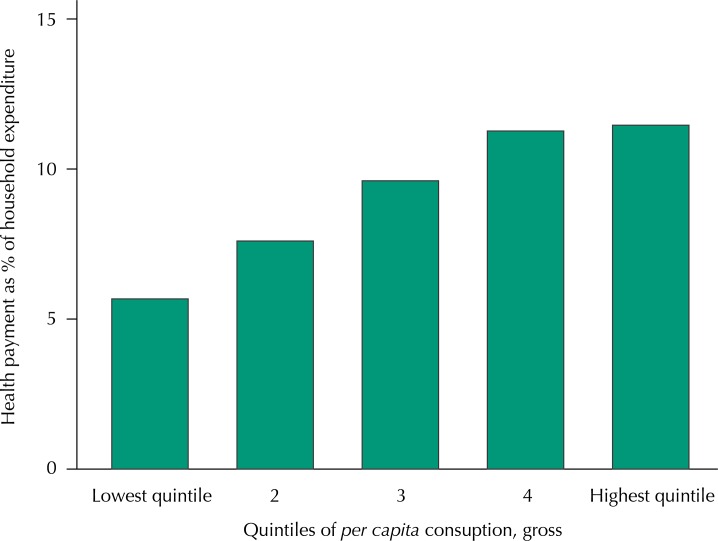
Out-of-pocket payments as a percentage of total household expenditure, per expenditure quintile.

The estimated Concentration Index is 0.5044 with standard error of 0.0121, and the Kakwani index is equal to 0.0717 with standard error of 0.0099. These two indexes add additional evidence of the progressivity of the system. Thus, the financial burden is concentrated in the wealthier population.

### Redistributive Effect

Table 4 presents the results from the decomposition of the redistributive impact of OOP payment on the healthcare financing system. The first part indicates that the poorest quintile is responsible for, on average 5.5% of total consumption, while the richest quintile consumes 49.5%. Similarly, the lowest quintile is consuming *per capita*, on average, 2.9% of OOP payment compared to the highest quintile, which consumes 54.1%. The wealthier contributed a much higher proportion of total OOP payment, while the last two quintiles contributed 77.1% of it. Overall, OOP payment represents an average of 10.4% of income.

**Table 4:** Decomposition of the redistributive impact of the healthcare financing system.

Variable	*Per capita* consumption, gross	OOP paymentfor healthcare
**%**	**%**
Quintiles of *per capita* consumption, gross		
Lowest quintile	5.5	2.9
Second quintile	9.5	6.9
Third quintile	14.2	13.0
Fourth quintile	21.4	23.0
Highest quintile	49.5	54.1
Total	100.0	100.0
Payments as fraction of Income (g)	1.0000	0.1038
Kakwani index assuming horizontal equity (Ke)		0.0616
Vertical effect (V)		0.0071
Horizontal inequality (H)		0.0031
Reranking (R)		0.0018
Total redistributive effect (RE = V - H - R)		0.0022
V/RE		3.2965
H/RE		1.4520
R/RE		0.8445

OOP: out-of-pocket

In the absence of horizontal inequity, the system is progressive; Kakwani index is 0.0616, indicating that the financial burden is concentrated in the wealthier population. The total redistributive effect of OOP payment has a positive sign, indicating reduction in inequalities and redistribution from the rich to the poor. The ratio of vertical effect over the redistributive effect is very large in comparison to the redistributive effect, indicating that redistribution is mostly a vertical effect, i.e., differential treatment of inequality. If there was no horizontal redistribution, then the vertical redistribution or Robin Hood role, i.e., taking from the rich to give to the poor, would be much greater. The vertical redistribution from rich to poor would be 230% greater than the current redistribution (Table 4), which indicates a considerable variation at different levels of income. The estimated *H* (a measure of horizontal inequity) is 0.0031 and it increases post-payment inequalities and reduces the redistributive effect. The differences in the composition of the population can help to explain this horizontal inequity. In conclusion, the rich contributes proportionally more than the poor to OOP payment, which can be attributed to their higher level of use of health services.

## DISCUSSION

Results from this study provide comprehensive understanding of the distribution of financial burden of the healthcare in Chile. Furthermore, our assessment of equity in the health system finance can identify policy variables for the ongoing debates and research in improving equity in the health system finance of Chile. Such findings, moreover, will also benefit other Latin-American Governments that are concerned about equity in their health system finance.

### Policy Implications

An interesting finding is that the health system finance in Chile is progressive, as judged by our estimation of OOP payment in relationship to income quintile. Therefore, persons with higher income are paying more OOP for healthcare than the poor. However, evidence of progressivity in the system does not mean that the system is equitable in all dimensions. In fact, a healthcare system may be progressive while still having serious issues of horizontal inequity^16^. We found evidence of horizontal inequity after examining the decomposition of the redistributive effect. It is at this point that we added the values of the 2PM for the analysis, since this model helps to identify some key variables that make the healthcare system more or less equitable.

The results from the 2PM showed an association between: age, education, insurance system, and the payment of the healthcare services. In addition, the heterogeneity in the sociodemographic status of the Chilean population may cause some differences and make the system less equitable. An interesting finding is that persons who are in the public insurance groups C and D and individuals with private system pay more OOP than persons in groups A and B. This is consistent with the principle of ATP, since groups A and B include the most disadvantaged persons; they are either indigent or individuals with very low income. We also found that persons from the FONASA groups C and D are paying similarly or more than those from ISAPRE. The tendency in the country is that persons with higher income have a propensity to belong to ISAPRE, which may contribute to the evidence of inequity that we observed in this study. This brings an additional policy issue: the availability of new subsidies to the healthcare system may favor ISAPRE beneficiaries instead of persons with lower income in the country. Allocation of public resources will be seen as inequitable under the actual structure. Therefore, the country should make additional efforts and reforms to promote better access by the poor to essential health care services.

The results of this study also indicate that an individual’s income is positively associated with his or her own financial burden of healthcare, which is driven mainly by the mixed public and private healthcare financing system in Chile. This means that, despite the incremental reforms towards more equitable health system finance in Chile, an individual’s income is still associated with the use of healthcare. Our study indicates that persons using the system more are those with a better economical position, which contradicts the higher healthcare needs that are often associated with lower income groups^10^. This indicates that a financial barrier might still exist for low-income groups to access healthcare in Chile. In order to improve financial access to healthcare for the low income group, Chile can implement a system mostly funded by the government using a progressive financing scheme, in order to achieve effective universal health coverage. Even though the Explicit Health Guarantees reform is an approach to provide universal and equitable health care coverage to the population, it still prevents the access of the population to health care services, imposing barriers and differential access for health conditions^4^.

Finally, based on our study, we suggest that there is an opportunity to examine the feasibility of providing greater access of the private healthcare delivery system for persons who belong to the lower quintiles. This is because while the government provides some healthcare facilities, they are often inadequate and, because of easier geographical and time access, persons use private provider facilities. To promote better access, especially for the low income group, the government can either expand its public facilities or subsidize the use of private facilities by low income groups. Similarly, if the system wants to improve the equity of healthcare financial burden, the government can increase public facilities and services, especially to make them available to disadvantaged groups. Besides improving public sector health services, evidence from our study and the literature suggest that improving an individual’s education can also contribute to a more equitable financial burden of healthcare and further equitable health^6^. Investment in general education, therefore, may also be a good long-term strategy to improve the health system finance equity of the country.

We cannot compare this study with previous empirical evidence. However, some comparative research reaches important conclusions about the status of OOP payment in the region. In many countries, most healthcare expenditures are financed by the government; however, OOP payment is still very important for the Latin American region. The World Bank, in 2006, undertook a comparative research in five Latin American countries finding that low public and high private healthcare expenditures characterize the Latin American and the Caribbean region, being most of it OOP (85%)^6^. Another study in the Caribbean region conducted by the Economic Commission for Latin America and the Caribbean in 2006 found a trend towards increasing dependence on OOP payment as a main source of health financing^8^. The OOP payment in countries such as Nicaragua or Guatemala finances more than half of their total national health expenditure, whereas in countries such as Chile and Argentina it represents no more than 25%^f^. Therefore, inequity in the financial burden of healthcare is one major concern for countries worldwide, including Chile.

### Limitations

The limitations are related to the use of secondary data. There is a long self-reported recall period of six to twelve months for most of the questions related to healthcare. In addition, we are aware that estimates of OOP payment can suffer from the same recall bias.

The 2006 Satisfaction and Out-of-Pocket Payment Survey did not ask for information related to the amount of payment for additional or voluntary health insurance, which may have an effect on the understating of the level of OOP payment of individuals. Moreover, our assessment was based entirely on OOP payment and did not include any other financial burden, especially taxation and insurance premiums.

The national survey data used had a 91.2% response rate and, as such, sample selection bias can be assumed to be minimal. Furthermore, the data is a national household survey that included both users and nonusers of healthcare services. Potential sample selection bias might occur in the second part of the ordinary least-square of the 2PM (i.e. ISAPRE beneficiaries might use more healthcare services and thus incur in more OOP payment). However, since we included the type of insurance as explanatory variable in both parts of the 2PM, sample selection bias, if it did exist, is minimized here given that sample selection bias is minimal in the entire national sample. The estimated coefficient for type of insurance provides the information on to what extent different types of insurance affect the probability of use versus nonuse of healthcare, and given the usage, the effect a particular type of insurance has on OOP payment. Additionally, we tested for endogeneity of the insurance variable using the following instruments: chronic diseases, main health problem, illness or accident, and type of home, so we performed a version of the Durbin-Wu-Hausman test, concluding that the null hypothesis that the variables are exogenous cannot be rejected.

Furthermore, results from the Concentration Index and Kakwani index indicate that, although higher income households proportionally contributed more with OOP payments, they also consumed more healthcare services than households with lower income. These results are evidence that, despite having health insurance, cost sharing may still be a barrier for the access to health services, especially by low-income households. Therefore, OOP payments could be a proxy measure for equity in the financial burden of healthcare. At the same time, we understand it is not a sufficient analysis of the overall healthcare equity, as it leaves out the equity analysis on the utilization of and access to healthcare.

## Footnotes

a WHO World Health Organization [cited 2017 Mar 9]. Available from: http://www.who.int/bulletin/volumes/85/2/06-037366/en/

b FONASA Fondo Nacional de Salud. Asegurados. Santiago de Chile; s.d [cited 2009 May 20]. Available from: https://fonasaweb.fonasa.cl/portal_fonasa/site/edic/base/port/asegurados.html

c Isapres de Chile. [cited 2011 Aug 5]. Available from: http://www.isapresdechile.cl

d Banco Central de Chile. Bases de dados estatísticos: indicadores diários [cited 2016 Feb 18]. Available from: http://si3.bcentral.cl/Indicadoressiete/secure/Indicadoresdiarios.aspx

e Superintendencia de Pensiones. Santiago de Chile; s.d [cited 2016 Feb 18]. Available from: https://www.spensiones.cl/

f WHO Statistical Information System. World health statistics 2009. Geneva; 2009 [cited 2009 May 30]. (Fact sheet, 290). Available from: http://www.who.int/whosis/whostat/2009/en
